# Monitoring Excavation-Induced Deformation of a Secant Pile Wall Using Distributed Fiber Optic Sensors

**DOI:** 10.3390/s25010254

**Published:** 2025-01-04

**Authors:** Chengyu Hong, Chengkai Xu, Weibin Chen, Jianwei Liu, Junkun Tan

**Affiliations:** 1 China Railway Seventh Group Co., Ltd., Zhengzhou 450016, China; cyhong@szu.edu.cn (C.H.); liujianwei79@163.com (J.L.); tanjunkunde@126.com (J.T.); 2 College of Civil and Transportation Engineering, Shenzhen University, Shenzhen 518060, China; jerrychenweibin@163.com; 3 Department of Civil and Environmental Engineering, The Hong Kong Polytechnic University, Hung Hom, Kowloon, Hong Kong, China

**Keywords:** BOTDA, distributed fiber optic sensors, bored pile wall, excavation

## Abstract

This paper investigates the use of the BOTDA (Brillouin Optical Time-Domain Analysis) technology to monitor a large-scale bored pile wall in the field. Distributed fiber optic sensors (DFOSs) were deployed to measure internal temperature and strain changes during cement grouting, hardening, and excavation-induced deformation of a secant pile wall. The study details the geological conditions and DFOS installation process. During grouting, the temperature increased by approximately 69 °C due to cement hydration 30 min post-grouting, while the strain decreased by 0.5% on average due to cement slurry shrinkage. During excavation, the temperature changes were minimal, but the excavation depth significantly influenced the strain distribution, with continuous compressive deformation observed in two monitored boreholes. Two analytical methods, the numerical integration method (NIM) and the finite difference method (FDM), were used to calculate the lateral pile displacement based on the monitored strain data. The results were compared with previous monitoring data, showing that the lateral displacement of the pile was minimal after excavation and was attributed to the high stiffness of the secant pile wall. This study demonstrates the effectiveness of DFOSs and BOTDA technology for monitoring complex pile wall behaviors during construction.

## 1. Introduction

In geological and geotechnical engineering, the excavation of cutting slopes disturbs the initial state of equilibrium of the soil mass and may lead to slope failure in the field [1,2,3,4]. In recent years, there have been slope failures triggered by a combination of excavation, continuous rainfall, and complex engineering geological conditions [5,6,7,8]. The retaining structures used to stabilize a soil mass include different types such as geosynthetic-reinforced soil walls [9], sheet pile retaining walls [10,11,12], prestressed anchor retaining walls [13], and bored pile retaining walls [14,15,16,17]. Among all these retaining structures, bored pile retaining walls have been demonstrated to be among the most effective structures for maintaining the overall integrity of slopes [14]. The excavation process has a significant influence on the stability of a soil mass, and a study on the mechanical performance of retaining structures during excavation is essentially required for rational field design and construction.

A variety of sensors have been developed for monitoring strain/stress distribution. To realize continuous measurements in space of the strain/stress characteristics of piles in large-scale geostructures, distributed fiber optic sensors (DFOSs) based on Brillouin Optical Time-Domain Analysis (BOTDA) or Brillouin Optical Frequency-Domain Reflectometry (BOFDR) have been used. For analyzing the end-bearing and skin friction behaviors and the deformation characteristic of piles utilizing DFOSs, a wide range of studies [18,19,20,21,22,23,24,25,26,27,28,29,30,31,32] were performed. However, these studies primarily focused on pile performance under loading conditions, paying limited attention to the strain and temperature variations during the cement grouting and hardening stages, as well as to the subsequent excavation process—factors that are critical for understanding the long-term stability of retaining walls. In addition, to evaluate the behavior of piles as retaining structures, DFOSs were utilized to obtain the full-length deformation along the pile depth produced by lateral soil movements, which was then used to calculate the lateral deflection of the piles based on numerical and analytical methods [16,17,33,34]. The lateral movement/displacement/deflection of piles can also be defined by the term “horizontal displacement”, which results from excavation or loading along one side of a pile wall. Nevertheless, most previous studies primarily focused on the introduction of new sensing technologies for monitoring the behavior of bored pile retaining walls [16], the effect of the excavation process on pile performance [16,17], and the performance of secant piled walls [33,34]. It could be concluded from the literature that, based on distributed BOTDA sensors, investigations on the assessment of full-length strain along bored pile retaining walls during the cement grouting and hardening processes are scarce. Furthermore, the analytical methods for the calculation of lateral pile movement have not been systematically discussed yet.

The traditional methods of monitoring DFOSs frequently necessitate lengthy periods of manual data reading in the field, and the effects of construction disturbances on the construction sites can cause DFOSs to receive high levels of noise, which leads to errors in the subsequent results and is an inherent challenge when DFOSs are used for construction monitoring. Recent advances in artificial intelligence provide opportunities to address such challenges in the field of DFOSs. Various machine learning techniques have been devised to improve the performance of DFOSs [35]. In addition, convolutional neural network models or algorithms, including the translation mean method [36], the Savitzky–Golay method [34,36,37], the percentile-filter method [36], and the Ransac algorithm [28], have been developed to perform real-time data denoising in BOTDA [38] and to accelerate the data processing efficiency of BOTDA [39]. A review of these methods will be useful in denoising the signals of DFOSs in investigations.

This study presents an investigation of the field monitoring of a bored pile wall subjected to excavation using DFOSs. An introduction of the BOTDA technique is first presented, and the sensors for field monitoring were calibrated. After geological investigations, these DFOSs were employed to measure the internal temperature and strain distribution profile of bored piles in the cement grouting, hardening, and excavation processes. Based on the measured strain distribution along the pile depth, lateral displacement was calculated by two analytical methods, and previous studies were also compared with the current study for a validation analysis. This study is innovative in systematically integrating the DFOS and BOTDA technologies to monitor the combined effects of cement grouting, hardening, and excavation-induced deformation, providing high-resolution, real-time strain and temperature profiles. Furthermore, by introducing two analytical methods to compute the lateral pile displacement, this research offers a robust alternative to traditional techniques, addressing critical gaps in understanding pile behavior during construction.

## 2. Brillouin Optical Time-Domain Analysis (BOTDA) Technique

The real-time monitoring of strain and temperature changes throughout the entire length of an optical fiber can be achieved through various DFOS techniques, including Optical Frequency-Domain Reflectometry (OFDR), Brillouin Optical Time-Domain Analysis (BOTDA), and Brillouin Optical Tim- Domain Reflectometry (BOTDR). Each of these techniques has unique advantages and limitations, making them suitable for different applications in monitoring large-scale piles during construction. BOTDA relies on the Brillouin interaction between a pulsed pump wave and a counter-propagating probe wave, where a local acoustic wave is formed at the intersection point and detected. However, this technique can fail if the optical fiber cable is disconnected due to harsh field conditions [40]. In such cases, the BOTDR technique can be used instead. In contrast to BOTDA, just a pulsed pump is delivered into the tested fiber from one side in BOTDR measurements. Since the time-resolved probe signal is back-reflected owing to spontaneous Brillouin scattering as opposed to stimulated Brillouin scattering, the signal strength is much lower than that obtained with BOTDA. The spatial resolution of BOTDA and BOTDR is often lower than that of OFDR (which is, e.g., ±1 cm in the sensing range of 50–70 m for Luna OBR 4600), and the OFDR technique can detect smaller changes in pile deformation [41,42]. However, this high resolution may also make OFDR more susceptible to the measurement noise caused by environmental factors, such as temperature fluctuations or mechanical vibrations, particularly in dynamic environments like construction sites. Additionally, the cost of the equipment and the complexity of data interpretation can be higher for OFDR than for the BOTDA and BOTDR systems, which are often more cost-effective for long-range and large-scale monitoring applications.

**Figure 1:**
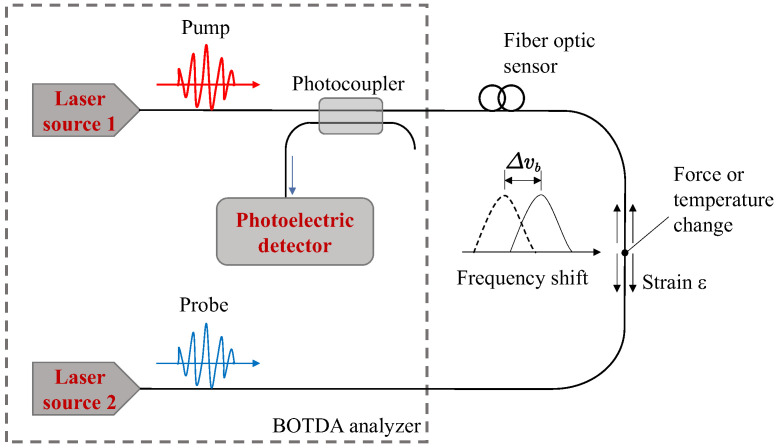
Stimulated Brillouin scattering (SBS) principle.

In the present study, BOTDA was used for measurements of both strain and temperature. The principle of the BOTDA technique is based on the stimulated Brillouin scattering (SBS) effect, as shown in Figure 1. A pump light pulse and a continuous spectrum are input into the two ends of a single fiber-optic sensor, and continuous light is amplified due to the Brillouin effect when the frequency difference between the two different light signals matches the Brillouin frequency shift. The axial strains and temperatures of fiber optic sensors can be directly measured on the basis of their linear relationship with the Brillouin light scattering (BLS) frequency change in the optical fiber. This linear relationship proposed by Horiguch et al. [43] can be written as:

(1) $$v_{B}\left(\varepsilon ,T\right)=v_{B}\left(\varepsilon_{0},T_{0}\right)+\frac{\partial v_{B}\left(\varepsilon ,T\right)}{\partial \varepsilon }\times \left(\varepsilon -\varepsilon_{0}\right)+\frac{\partial v_{B}\left(\varepsilon ,T\right)}{\partial T}\times \left(T-T_{0}\right)$$

where $v_{B}\left(\varepsilon_{0},T_{0}\right)$ and $v_{B}\left(\varepsilon ,T\right)$ are the frequency shift of Brillouin scattered light before and after the test, and $\varepsilon_{0}$ and ε are the axial strain at a certain spatial resolution of the optical fiber sensor before and after the test, respectively. $T_{0}$ and T are the surrounding temperature before and after the test. $\frac{\partial v_{B}\left(\varepsilon ,T\right)}{\partial \varepsilon }$ and $\frac{\partial v_{B}\left(\varepsilon ,T\right)}{\partial T}$ are the calibrated strain coefficient and calibrated temperature coefficient.

The field monitoring test utilized a DiTeSt SN0050 Dual Reading Unit data logger manufactured by Omnisens (Switzerland) [44], which has a sampling resolution of 0.2 m and a spatial resolution of 0.5 m. The measurement accuracy of the strain and temperature measurements was at a level of ±5 με and ±0.2 °C, respectively [16]. The frequency step was set to 2 MHz, and the reading interval was 2 ns during the measurement. In the field, a single measurement took an average of 87 s, and the signal from each fiber was read twice to minimize random errors. The BOTDA settings information is summarized in Table 1.

## 3. Laboratory Calibrations

**Figure 2:**
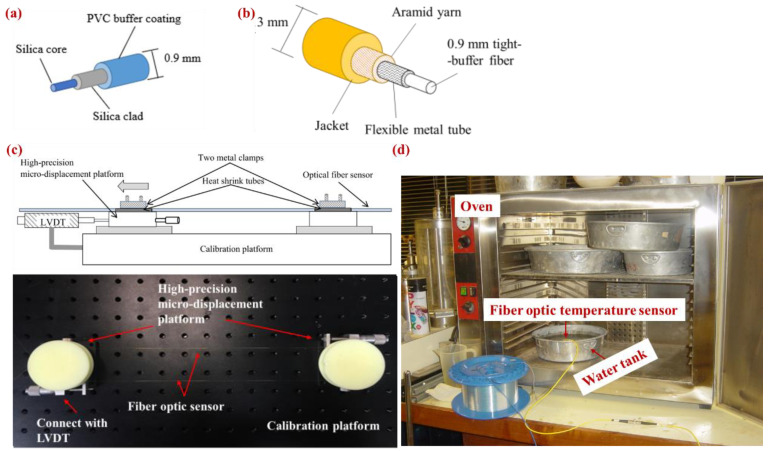
Calibration test: (**a**) structure of bare FOS for strain measurement, (**b**) temperature compensation fiber, (**c**) overall view of strain calibration setup, and (**d**) overall view of temperature calibration setup.

In this study, two kinds of easy-bending single-mode fibers (SMFs) were adopted for the strain and temperature measurements. Figure 2a shows the structure of a bare FOS for the strain measurement of piles. The external diameter of the strain sensor was 0.9 mm. The SMF for strain measurement not only provides improved performances with extremely low fiber attenuation at wavelengths ranging from 1260 nm to 1625 nm, but also has excellent bending resistance because it was manufactured by a plasma-activated chemical technique. To improve the fiber’s longevity, the SMF was coated with two layers of UV-cured acrylic including a silica clad and a PVC buffer coating. An armored optical cable was used not only for signal transmission but also for temperature monitoring, as shown in Figure 2b. This temperature sensor cable mainly includes a 0.9 mm tight-buffer fiber (for temperature monitoring), a flexible metal tube, an aramid yarn, and an external jacket (3 mm). The SMF for the temperature sensor is loosely placed into a hollow steel tube. The inner fiber is loose and shielded by the metal tube, allowing it to be solely exposed to temperature changes. As mentioned by Soga and Luo [41], there is a large space for movement between the metal tube and the fiber because the 0.9 mm tight-buffer fiber is loose inside the metal tube. Therefore, it can be seen that the strain information is not transmitted through the metal housing to the internal fibers. In summary, the fiber has a high strain sensitivity and can withstand extreme environmental conditions.

**Figure 3:**
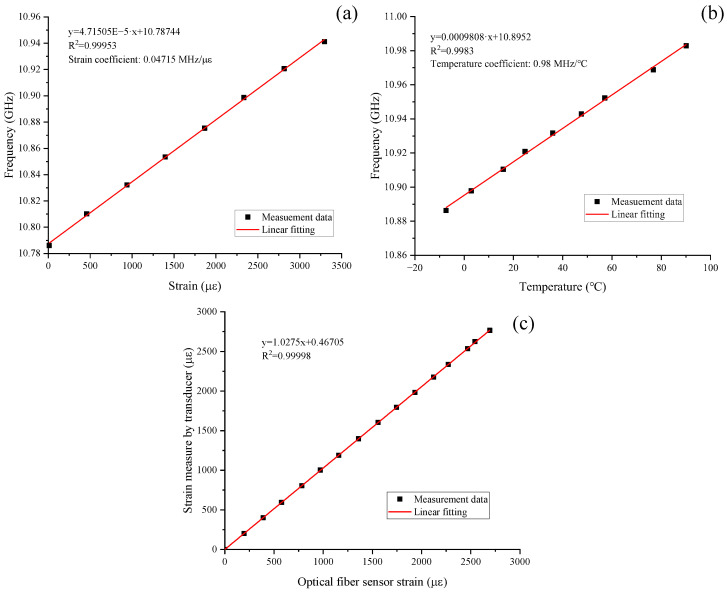
Calibration results of the DFOS: (**a**) calibration results of the DFOS for strain measurement, (**b**) calibration results of the DFOS for temperature measurement, and (**c**) comparison of strain measured by LVDT and DFOS.

A series of laboratory calibration experiments were carried out by exerting varied strains on a specified length of the SMF. Figure 2c shows an overall view of the setup of the calibration test. The SMF cable was mounted on a calibration platform and twisted into six loops between two high-precision micro-displacement platforms with a diameter of 40 mm and a spacing of 350 mm, whereas one of the micro-displacement platforms was coupled with a linear variable differential transformer (LVDT) with measurement accuracy of 0.01 mm to control the applied strain and to verify the calculated strain. As shown in Figure 3a, the Brillouin frequency shift had a highly linear relationship with the applied strain (interval of 500 micro strains), and we could obtain the strain coefficient of 0.04715 MHz/με through this relationship. The calibration result for the temperature is shown in Figure 3b, which also shows a clear linear relationship between Brillouin frequency shift and temperature. Based on the obtained strain coefficient, the calculated strain was verified by the measured strain by the LVDT. As shown in Figure 3c, the calculated strain agreed well with the measured strain. In this study, the structure of the fiber optic cables used in the calibration tests was the same as that of the inner bare fiber optic cable for field monitoring applications, except for one external PVC protection layer. Based on previous literature reporting that the strain transfer coefficients from the monitored substrates to fiber optic sensors are mostly between 0.95 and 0.99, it appears that almost all strains of substrates can be transferred to inner bare fiber optic strain sensors [45,46,47]. In addition, the fiber optic was placed in a sonic tube commonly used in cast-in-place piles and grouted into an integrated section by cement [48]. Similar to the situation in this study, where a dual-layer-coating fiber embedded in cement withstood a uniform strain field, 95% of the solid strain could be developed in the fiber beyond 55 mm, and 99% of the strain could be developed over 84 mm from both ends of the cable [47]. Therefore, the influence of the external protective layer on the strain transfer coefficient for transfer between the monitored substrate and the fiber optic sensor was limited (less than 5%).

## 4. Engineering Geological Condition and Field Monitoring

### 4.1. Engineering Geological Condition

**Figure 4:**
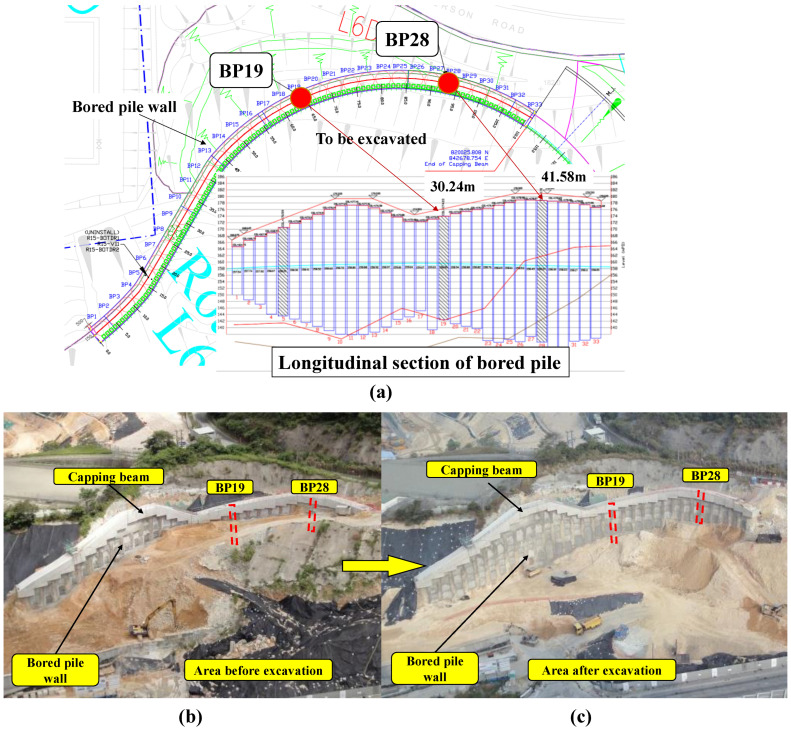
Overall view of the field site: (**a**) schematic view of the locations of the instrumented piles, (**b**) photo of the site before excavation, and (**c**) photo of the site after excavation.

It is necessary to conduct engineering geological investigations, which are fundamental for the selection of construction sites and the reinforcement design for slopes subject to excavation investigations. The monitoring project was located at Anderson Road in Sai Kung District, east coast of Hong Kong. This district is a seaside region known for its landscape, little settlements, and beautiful seascapes. A traffic road was constructed at the bottom of the soil slope, and the upslope was a service reservoir facility. A secant pile wall was constructed to stabilize this soil slope, which was subjected to multi-stage excavations. This retaining wall includes 33 bored piles, reaching 3.0 m in diameter for each pile. A capping beam with dimensions of 2.0 m in height and 3.0 m in width was constructed to connect all bored piles. A schematic view of the secant pile wall is shown in Figure 4a. Photos of the site in this study before and after slope excavation are shown in Figure 4b,c.

**Figure 5:**
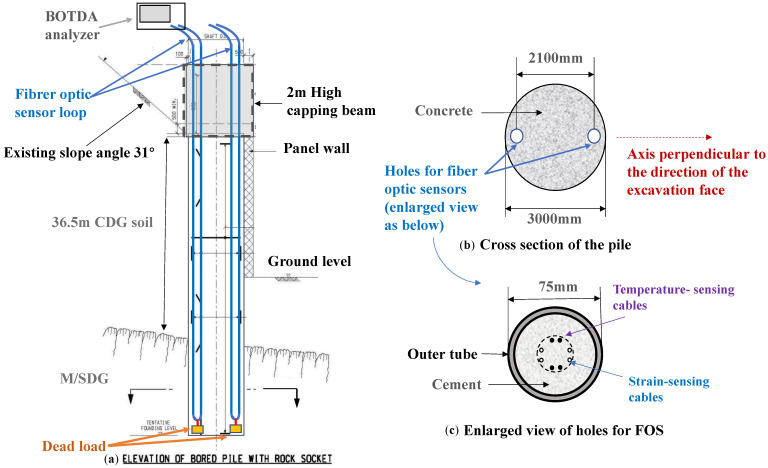
Schematics of geological profile and DFOS layout scheme: (**a**) overview of field monitoring, (**b**) cross section of a pile, and (**c**) detailed location of FOS.

According to the geological survey report, the geological profile around the secant pile wall is shown in Figure 5a, with a 36.5 m thick layer of completely decomposed granite (CDG) covering moderately to slightly decomposed granite (M/SDG). Some physical properties of CDG are summarized in Table 2. The slope surface was inclined at 30° with respect to the horizontal. It should be noted that the heterogeneity of the CDG layer, including local fractures and variations in material properties, may influence the performance of DFOSs. For example, localized discontinuities or weak zones within the soil could lead to irregular signal fluctuations or noise in the strain measurements. To mitigate these potential impacts, we optimized the sensor layout and employed advanced data processing techniques. Two bored piles (BP19 and BP28) with length of 30.24 m and 41.58 m (as shown in Figure 4) were mounted with DFOSs in this study to investigate the mechanical behavior of piles subjected to excavation. In each monitoring pile, two prefabricated holes were reserved for steel tubes for the installation of DFOSs, as illustrated in Figure 5b. A total of four SMF cables, including two temperature-monitoring cables and two strain-monitoring cables, were installed in two opposite holes, perpendicularly to the direction of the excavation face of the secant pile wall, as shown in Figure 5c. The temperature-monitoring fibers were installed within rigid sleeves, allowing them to slide freely and remain unaffected by strain, ensuring that their signals only reflected temperature variations. The strain-monitoring fibers, on the other hand, were bonded to the structure and thus subjected to both strain and temperature effects. By using the temperature data from the temperature-monitoring fibers for compensation, the temperature effects on the strain-monitoring fibers were removed, isolating the strain-induced signal. The distance between the two opposite holes was 2100 mm. This configuration was carefully designed to capture both tensile and compressive strains along the pile depth induced by the excavation, as well as to monitor temperature variations during cement grouting and hardening. The placement of the holes perpendicularly to the excavation face allowed the sensors to effectively measure the bending behavior of the pile under asymmetric soil pressure and excavation-induced deformation. Additionally, embedding the cables in opposite holes ensured symmetrical monitoring, minimizing potential measurement errors caused by construction disturbances or uneven loading conditions. Furthermore, this layout was determined based on the pile diameter, as the installation of too many sensors could result in mutual interference, reducing the measurement accuracy. Temperature sensors and strain sensors were installed in close proximity to enable temperature compensation for the strain measurements, thereby improving the reliability and precision of the strain data. This balanced approach ensured sufficient data resolution while avoiding overcrowding of the sensors, addressing both technical constraints and monitoring objectives.

### 4.2. Field Monitoring

**Figure 6:**
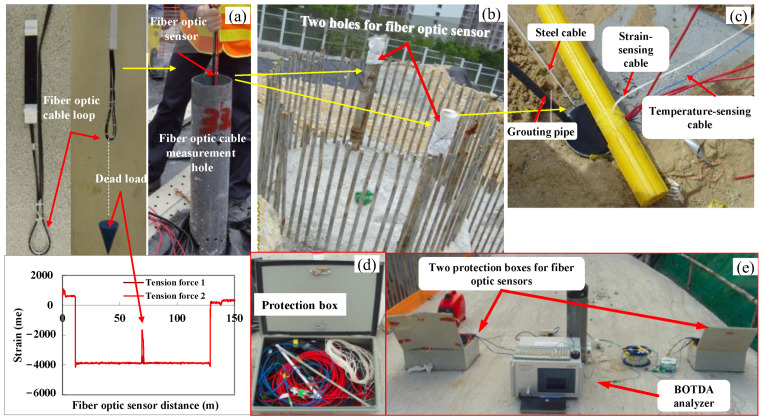
Field installation and monitoring of the DFOSs with the BOTDA sensing technology: (**a**) central section of each SMF cable suspended by a dead load, (**b**) location of the reserved tubes, (**c**) installations of all cables, (**d**) protection box for the pile embedded with DFOSs, and (**e**) field work of data collection and reading using the BOTDA analyzer.

Some details of the field installation of the DFOSs in this case are shown in Figure 6. When designing the spacing of the two fiber optic sensors, in order to sufficiently measure the strain when the pile is bending, the measurement points must be symmetrically arranged at a certain distance from the center of the pile. This is because it is difficult to ensure uniformity of the concrete–soil contact interface when a bored pile is introduced, and the steel reinforcement placed in the pile may lead to an uneven strain distribution in the pile. Therefore, the design of the monitoring system was based on the expected maximum horizontal displacement of 20–60 mm prior to construction and the average Young’s modulus E_0_ = 28 × 106 kPa of the bored pile. At a spacing of 2100 mm (as shown in Figure 5b), the maximum strain was between 0.3 and 0.6%, which falls within the practical range for fiber optic strain monitoring that has an upper strain limit of approximately 3–5% [49]. Figure 6a shows that the central section of each SMF cable was suspended by a dead load, which kept the cable vertical and confirmed the bottom location of the cables when reading the sensing data. Each DFOS cable was connected in a measurement loop and placed into the reserved tubes, as shown Figure 6b. Imai et al. [50] agreed that it is feasible and advantageous to install DFOSs inside the structural components, for example, by embedding them inside the concrete, which has numerous advantages. Likewise, the DFOSs were embedded inside the structural components, as shown in Figure 6c.

It is worth noting that the whole monitoring hole was filled with cement grout, which ensured that all fiber optic sensors successfully reflected the pile wall deformation after the cement hardened [47,48]. Figure 6d shows two protection boxes for the placement of DFOSs. Figure 6e illustrates the setup of data collection from fiber optic sensors using the BOTDA analyzer. According to Kechavarz, et al. [51], it is also crucial to have some prior knowledge of the size of the minimum strain that has to be recorded and to make sure that it is much larger than the measurement performance in order to improve the performance of the developed system in the field load test. For a secant pile wall, the initial maximum axial strain can be estimated with prior knowledge of the applied force and pile diameter.

## 5. Monitoring Results and Analysis

### 5.1. Data Reading and Processing

**Figure 7:**
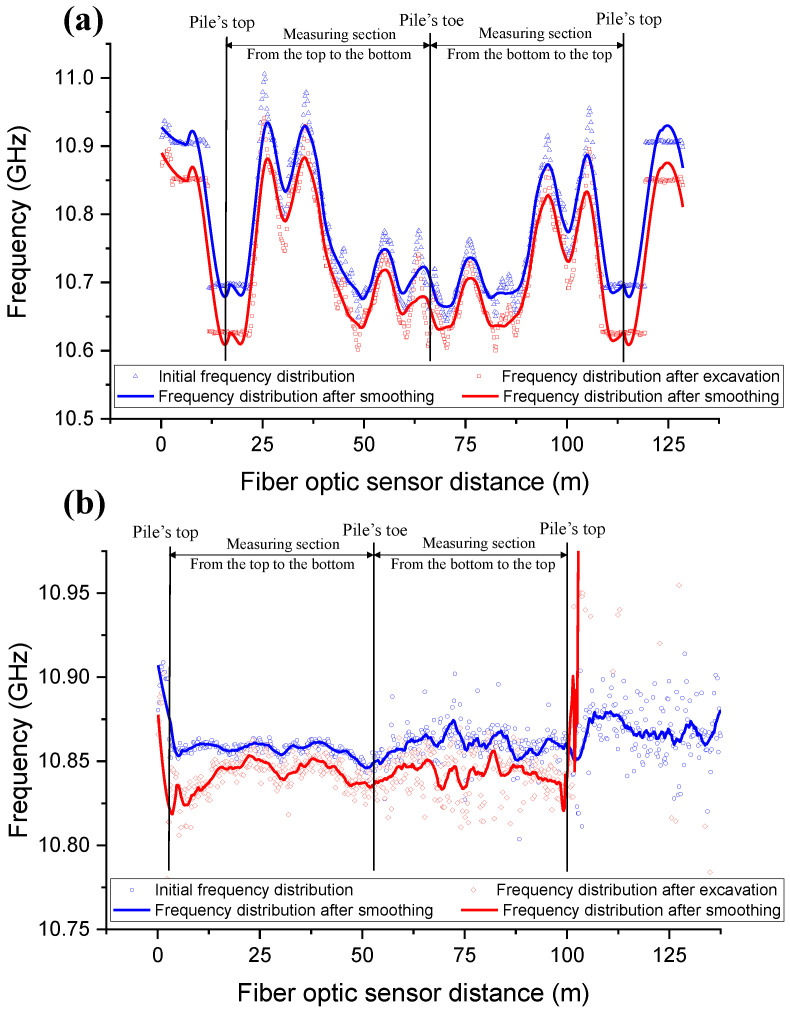
Initial and smoothed frequency distributions along the DFOS for (**a**) stain monitoring in pile BP28 and (**b**) temperature monitoring in pile BP28.

Figure 7 illustrates the initial frequency distributions for stain monitoring and temperature monitoring of the pile BP28. The measured data can be separated into two symmetrical measurement sections from pile head to pile toe, representing a loop of the sensor cable along the pile depth. The obtained initial frequency distributions showed substantial fluctuations. Previous research has shown that the data analysis of distributed strain/temperature measurements obtained in the field has always been complex due to unavoidable measurement errors produced by the complex strain input as well as the analyzer’s performance restrictions [52,53]. To make an accurate judgment on the deformation of a pile, it is necessary to analyze the original data and undertake smoothing analysis using scientific procedures. Various scientific approaches, at present, have been used to process the original signal, including the translation mean method [36], the Savitzky–Golay method [34,36,37], the percentile-filter method [36], and the Ransac algorithm [28]. Rolling filtering is a technique for eliminating noise from measurement data [52]. As the BOTDA analyzer evaluates a weighted average of strain across space, the Savitzky–Golay filter [54] was considered for noise reduction. This method fits a polynomial to a local subset of data points within a defined window, minimizing the least-squares error. The filter parameters, including the polynomial order and the window size, significantly influence the results. Larger window sizes result in excessive smoothing and loss of detail, while higher polynomial orders introduce oscillations that are not representative of the physical strain distribution. A second-order polynomial with a window size of 15 was selected after testing different parameter combinations on a subset of the data. This configuration has been shown in prior research to effectively reduce noise while maintaining the physical characteristics of distributed strain and temperature profiles [34,36].

The comparisons between the original frequency distributions and the smoothed distributions are also presented in Figure 7. It is clear that the fluctuation amplitudes of the raw distributions are reduced, and all curves become smoother as a result of the filtering of unnecessary noise and surrounding interferences. In addition to the introduction of a smoothing analysis, the two symmetrical measurement sections were averaged. The smoothed and averaged frequency distributions can be used to compute both strain and temperature distributions using previous calibrated coefficients, as described in Section 3. Both smoothed and averaged strain distributions (pure strain change) were obtained considering the measured temperature distribution using the fiber optic sensors. The temperature difference between the two measurements was calculated and compensated in relation to the strain value by the temperature compensation factor of 18.793 με/°C provided by Omnisens, Switzerland [44].

### 5.2. Effect of Grouting and Cement Hardening

**Figure 8:**
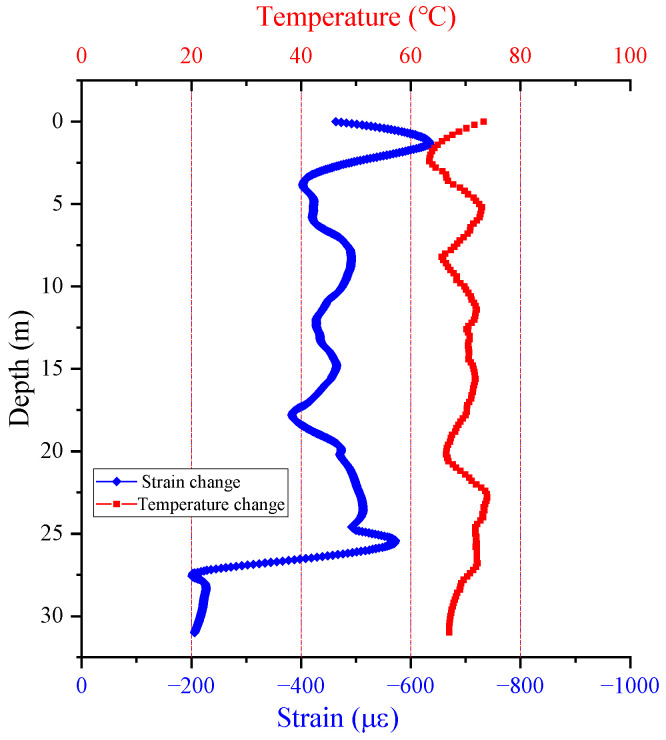
Changes in strain and temperature in two data collections (once grouting was completed and over the complete grouting of 30 min) along the length of BP19.

In the grouting process, it is inevitable that the flow of cement slurry will affect the position of the FOS cables, causing errors in the measured data masking the real effects of the cement slurry. Therefore, first, sensor data were collected immediately once grouting was completed, and a second data collection was conducted over the complete grouting of 30 min. The strain and temperature distributions obtained in the two data collections for BP19 are shown in Figure 8. It can be seen that the temperature increased in a range from 63 to 73 °C (about 69 °C on average), while the obtained shrinkage strain was within the range of 0.2–0.6% (around 0.5% on average) 30 min after grouting was completed. The temperature fluctuated with the pile depth, approximately ranging from −5 °C to +5 °C. Similar fluctuations occurred in some previous studies [24,55]. It was inferred that this fluctuation was mainly caused by the following factors: (1) the buried piles were surrounded by CDG and M/SDG, or the CDG stratum was heterogeneous (as shown in Figure 5a); (2) there might be unavoidable cracks or defects within the pile after construction. The crack theory is based on the concept that cracks are essentially structural discontinuities that may alter the stress and strain distribution inside a material. The existence of fractures may lead to localized stress concentrations, which in turn might cause fluctuations in the strain signals [56]; (3) construction disturbances on construction sites can cause DFOSs to receive amounts of noise that can lead to errors in the subsequent results. During the grouting process, the heat emitted by the cement hydration reaction rapidly leads to a temperature rise in the cement slurry, but rapidly dissipates in the following half hour. Such a high hydration temperature (60–80 °C) was also reported by Fellenius et al. [57]. The strain change mainly resulted from the shrinkage deformation of the cement slurry in the process of the hydration reaction. The cement slurry at the pile tip started the hydration reaction earlier, since cement grouting was conducted from the pile tip to the top. As a result, the shrinkage degree at the bottom was relatively smaller than at other positions. It may be worth noting that the uncorrelation between temperature and strain in this study (as shown in the following Figure 8) was proved by a Spearman’s correlation coefficient ρ of −0.083, which is larger than −0.4. Kowalczyk et al. [58] assumed that two variables are correlated only when ρ is in a range from −1 to −0.4 and from 0.4 to 1, indicating that the presented temperature sensor is insensitive to strain.

**Figure 9:**
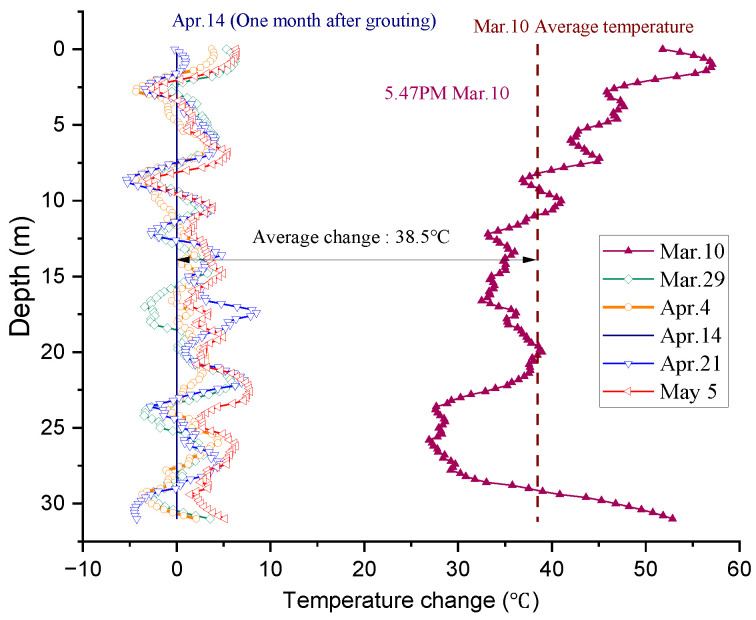
Average temperature profile measured from March 10 (after grouting, before initial setting time of cement) to May 5 along pile BP19.

The temperature distribution profile of BP19 measured after cement hardening (April 14) was used as the baseline, and the temperature change along the pile, measured from March 10 (after grouting, before the initial setting time of the cement) to May 5, is plotted in Figure 9. As shown in Figure 9, the average temperature measured on the day of grouting was higher than that measured one month later, in the range from 27 °C to 54 °C (38.5 °C in average), which was due to the heat concentration and dissipation caused by the hydration reaction of the cement grout. After the initial setting time of the cement grout, the measured temperature profile varied within a limited range (from −5 to 5 °C), indicating that the hydration reaction was completed after March 29. This observed temperature variation with the pile depth was mainly attributed to a temperature change in the surrounding environment.

**Figure 10:**
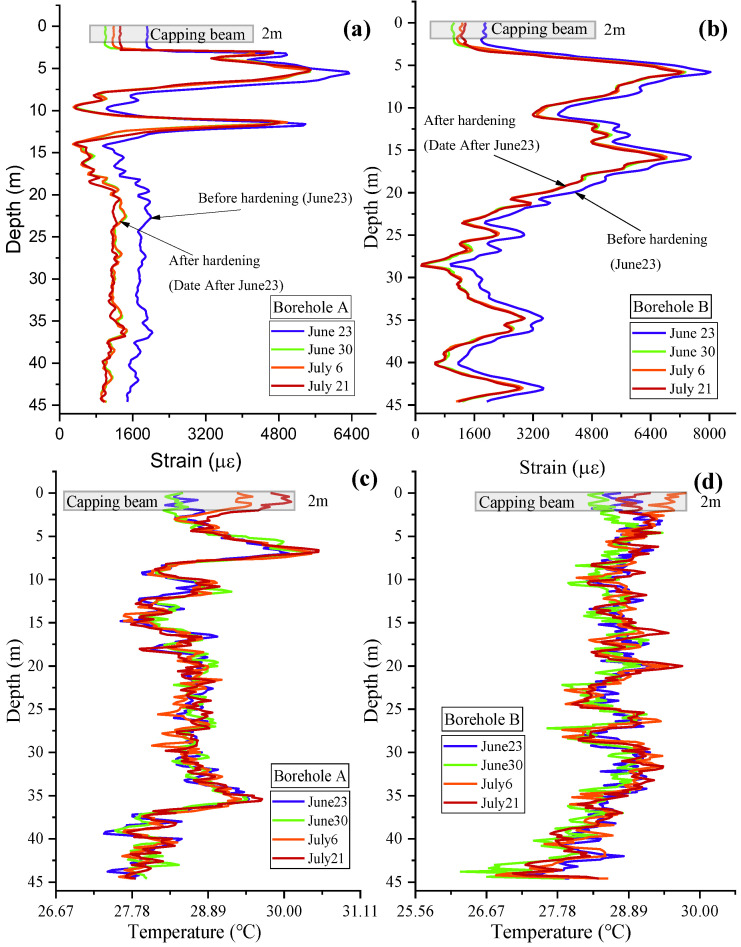
Strain and temperature distributions in two boreholes of BP28 before and after hardening of cement grout: (**a**) strain profile of borehole A, (**b**) strain profile of borehole B, (**c**) temperature profile of borehole A, and (**d**) temperature profile of borehole B.

Before and after cement hardening, the strain and temperature distributions in two boreholes of BP28 were measured to evaluate the effect of the hardening process on the pile performance, as shown in Figure 10. It is seen in Figure 10a,b that an obvious strain reduction before and after hardening was clearly observed. Both strain distributions measured at deeper positions close to the pile tip were relatively lower than the strain at lower positions. The strain distributions inside two capping beams were similar, presenting consistent variation trends as time elapsed. The temperature distribution showed a slight change after the hardening of the cement grout, as shown in Figure 10b,c. The temperatures measured at the pile tips were both around 2.5 °C lower than at the pile head.

### 5.3. Effect of the Excavation Progress

**Figure 11:**
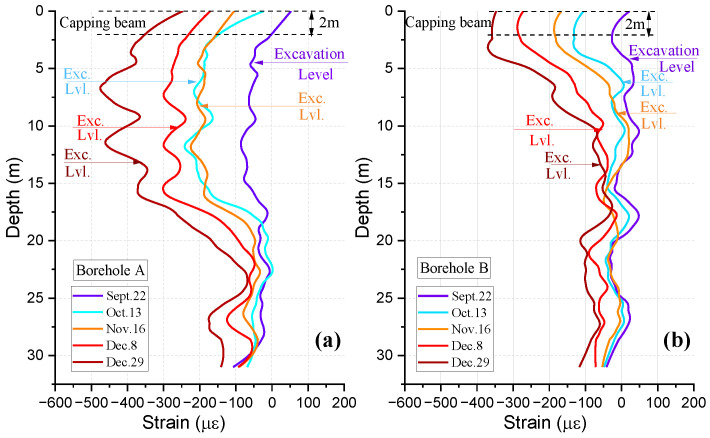
Measured strain profiles along pile BP19 at different excavation stages for (**a**) borehole A and (**b**) borehole B.

Both initial temperature and strain distributions before the excavation were used as reference baselines to investigate the influence of different excavation stages on the deformation characteristics of the pile. Figure 11 depicts the strain distribution profiles in the two opposite boreholes of BP19 and the corresponding excavation depths at different excavation stages (From September 22 to December 29). Compressive strain distributions inside the two boreholes were clearly observed and presented a significant increment in magnitude as the excavation progressed. The excavation depth had a significant influence on the strain distribution profiles of pile BP19. The strain distribution inside the borehole close to the excavation area (within a range from −450 με to 0 με, as shown in Figure 11b) was higher than that inside the borehole away from the excavation area (within a range from −380 με to 0 με, as shown in Figure 11a). The magnitude of the compressive (negative) strain increased as the excavation depth increased, and the maximum negative strain moved downward as the excavation progressed from 4 m to 13 m.

**Figure 12:**
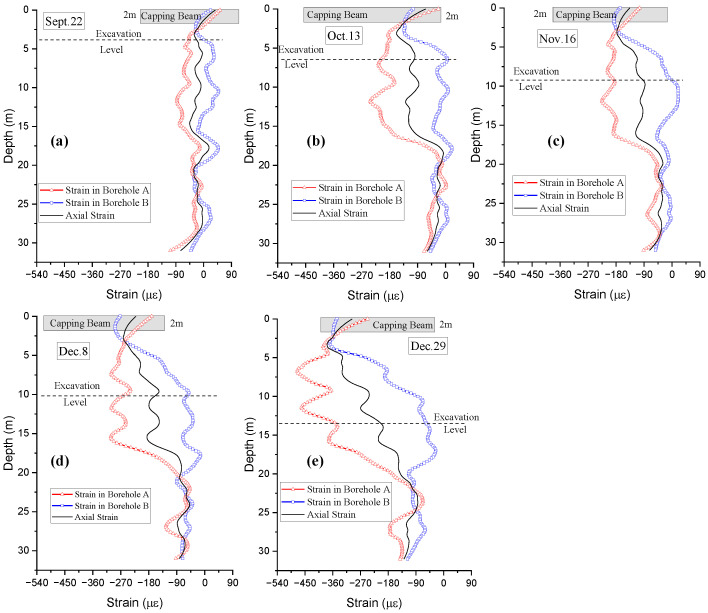
Comparison among strain distributions in two boreholes of BP 19 and their average strain distributions at different excavation levels: (**a**) September 22nd; (**b**) October 13th; (**c**) November 16th; (**d**) December 8th; (**e**) December 29th.

Figure 12a–e depict the strain profile distributions inside two opposite boreholes of BP19 at different excavation dates or excavation levels (excavation depths). The obtained averaged strain clearly indicates that the bored pile was under substantial compression. The larger compressive strain at borehole A (close to the excavation area) indicates that the pile moved toward the excavation face. All curves for the strain inside the two boreholes intersect at the bottom of the capping beam (2 m underground), revealing that the capping beam constrained the pile movement during the excavation.

## 6. Analytical Methods for the Calculation of the Lateral Pile Movement

**Figure 13:**
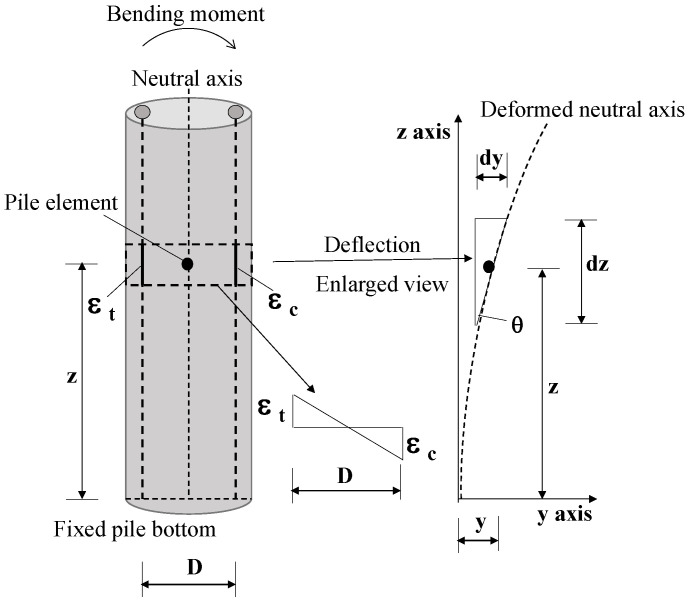
Schematics of deflection calculation for beam element.

It is essential to investigate the lateral deflection of piles caused by soil excavation and the location of the maximum lateral deflection. In field monitoring, the conventional point inclinometer was popular and extensively applied to measure lateral displacement [16,33,37,59,60,61]. Alternatively, based on the strain distribution observed with DFOSs, two numerical approaches, including the finite difference method (FDM) and the numerical integration method (NIM), were successfully used to compute lateral displacement against pile depth [16,62]. The NIM is a popular method to obtain the deflection y(z) of piles with the assumption that strain distribution along the longitudinal direction of a pile is linear, as shown in Figure 13. This assumption aligns with the basic beam theory, and the relationship between curvature and strain is expressed as:

(2) $$y^{\prime \prime }\left(z\right)=k=\frac{M}{EI}$$

(3) $$\frac{M}{EI}\times \frac{D}{2}=\frac{\varepsilon_{t}-\varepsilon_{c}}{2}$$

where M, E, and I are bending moment, elastic modulus, and moment of inertia of the cross section of a pile, respectively; k is the curvature coefficient; D is the distance between two opposite fiber optic sensors; and $\varepsilon_{t}$ and $\varepsilon_{c}$ represent the tensile and the compressive strain, respectively. Combing Equations (2) and (3) yields:

(4) $$y(z)=\frac{1}{D}\iint (\varepsilon_{t}-\varepsilon_{c})dzdz$$

To calculate the standard deviation of the displacement using the NIM, we need to integrate the square of the strain differences over the length of a pile. For the 95% confidence interval, the accuracy of the displacement is approximately calculated as 0.155 mm.

The FDM discretizes the governing equations of the basic beam theory into a finite difference form, allowing the curvature and displacement to be calculated iteratively over small segments of the pile. It is assumed that a continuous beam is separated into several tiny elements, and the interval between adjacent elements is assumed to be *h*. The first derivatives of y at the locations z−h, z, and h are obtained as follows:

(5) $$y^{\prime }\left(z\right)=\frac{1}{h}[y\left(z+h\right)-y(z)]$$

(6) $$yy^{\prime }\left(z-h\right)=\frac{1}{h}[y\left(z\right)-y(z-h)]$$

where $y^{\prime }\left(z\right)$ and $y^{\prime }\left(z-h\right)$ are the slopes of the deflection of the pile depth from z to z+h and from z−h to z, respectively. The second-order derivative can be obtained as shown below:

(7) $$\begin{array}{r} y^{\prime \prime }\left(z\right)=\frac{1}{h}\left\{\frac{1}{h}\left[y\left(z+h\right)-y\left(z\right)\right]-\frac{1}{h}\left[y\left(z\right)-y\left(z-h\right)\right]\right\}\\ =\frac{1}{h^{2}}\left[y\left(z+h\right)-2y\left(z\right)+y\left(z-h\right)\right] \end{array}$$

To calculate the overall pile deformation from discrete strain measurements distributed along the pile length based on the elasticity theory, it is assumed that the pile deflection is small and the strain distribution along pile section is linear. This assumption was also made in previous studies [34,63,64]. According to the basic beam theory, we can use Equations (2) and (3) combined with Equation (7) to obtain the lateral displacement of the pile:

(8) $$yy\left(z+2h\right)-2y\left(z+h\right)+y\left(z\right)=\frac{h^{2}}{D}\left(\varepsilon_{t}-\varepsilon_{c}\right)$$

Equation (8) can be solved by the given boundary conditions. In this study, the monitored pile was inserted into slightly decomposed granite; so, the pile tip could be considered as a fixed end.

**Figure 14:**
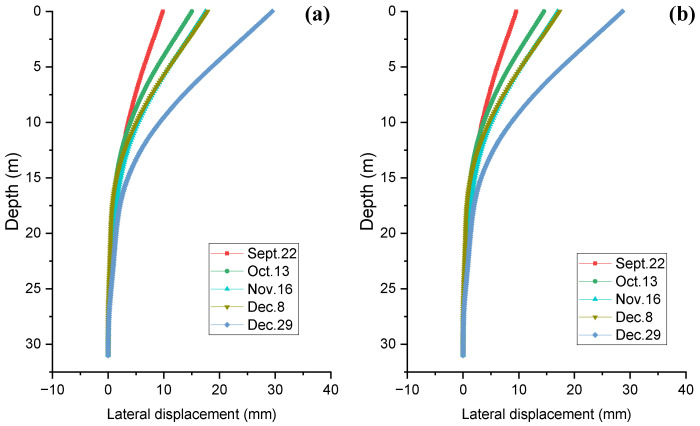
Comparison between the lateral displacements calculated by (**a**) NIM and (**b**) FDM.

Figure 14 shows the lateral displacement distributions calculated by both NIM and FDM at five different excavation stages. The two lateral displacement distributions along the pile depth at different excavation levels calculated by NIM and FDM were consistent, with a maximum difference of less than 3% being obtained at the pile head. The lateral displacement increased from the pile tip to the pile head, approaching a maximum level at the pile head. The maximum lateral displacement increased as the excavation work proceeded. In addition, within a 95% confidence interval, the true value of displacement was within ±0.155 mm of the calculated result.

## 7. Discussion

**Figure 15:**
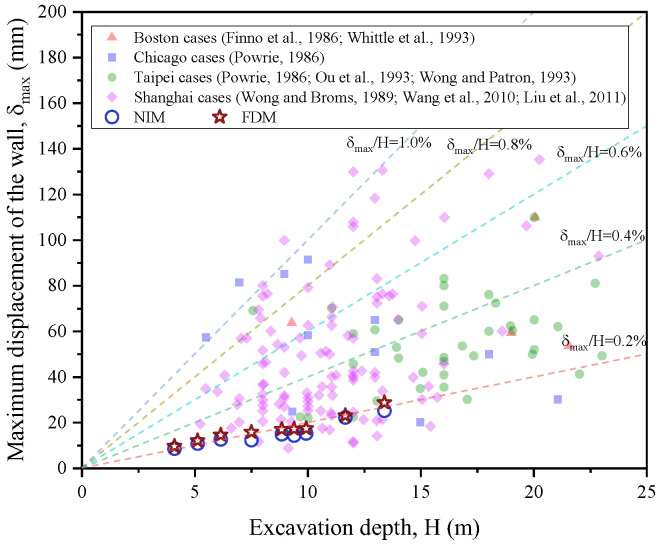
Comparison of maximum lateral deflection versus excavation depth between the current study and previous studies [65,66,67,68,69,70,71,72].

To further verify the calculated lateral displacement based on NIM and FDM, some relevant studies [65,66,67,68,69,70,71,72] were selected for comparison. Figure 15 shows the maximum lateral displacement against the excavation depth from the present study and some previous studies. The maximum displacement of the presented secant pile wall obtained by NIM and FDM increased as the excavation depth rose, which is consistent with the curve trends observed in previous studies. The ratio of the maximum displacement to the excavation depth in the present study was around 0.2%, which is relatively low compared to the values obtained in previous studies. This difference may be attributed to the fact that the maximum deflection is also influenced by retaining stiffness, soil strength, water level change, and construction methods. To objectively discuss the relationship between maximum lateral displacement and excavation depth, Spearman’s correlation coefficient ρ was also used to evaluate the strength and direction of the monotonic association between maximum lateral deflection and excavation depth. In this study, the calculated ρ between maximum lateral deflection and excavation depth equaled 0.423, which is larger than 0.4 (as mentioned in Section 5.2). This fact reconfirmed that the maximum displacement had a positive correlation with the excavation depth.

**Figure 16:**
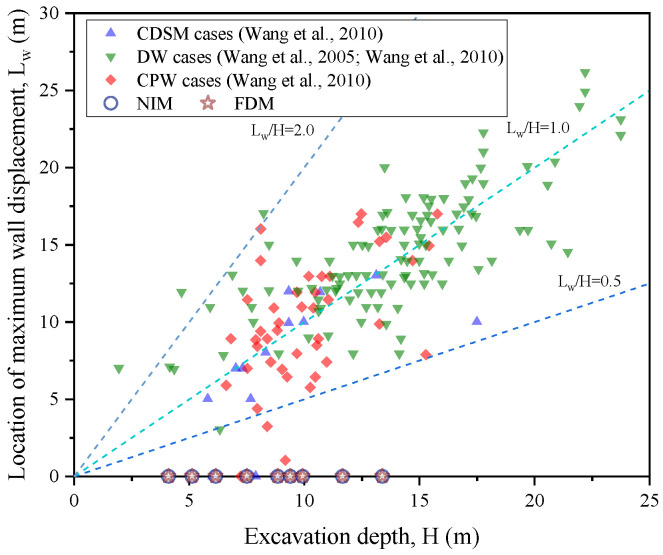
Comparison of the locations of maximum wall displacement versus excavation depth between the current study and previous studies [71,73].

Figure 16 presents the locations of the maximum wall displacement against the excavation depth from the present study and previous studies [71,73]. The previous studies were divided into three groups according to the type of the retaining walls (including compound deep soil mixing walls, diaphragm walls, and contiguous pile walls). In most previous studies, the maximum deflections were present when the ratio of L_w_/H was within the range of 0.5–2.0. The ratio of L_w_/H in the present study was limited (less than 0.1), indicating that the stiffness of the secant pile wall was high, and the influence of the excavation work on the pile movement was ignorable.

However, the deformation behavior of a pile during excavation is governed by the soil–structure interactions, which are influenced by the excavation depth, the soil properties, and the pile characteristics. As the excavation depth increases, the removal of lateral soil support induces stress release and lateral soil movements, which impose bending moments and shear forces on the pile. The pile resists these movements through its stiffness and embedment depth, which results in a coupled soil–pile response. The results presented in this study, which analyzed the effect of excavation depth on pile deformation, are consistent with these soil–structure interaction mechanisms. As the excavation depth increased, the lateral soil movements became more pronounced, exerting greater forces on the pile and resulting in larger displacements.

Moreover, compared to traditional monitoring methods (e.g., inclinometers, strain gauges, extensometers), DFOSs offer distributed measurements with high spatial resolution (millimeters to centimeters), enabling the detection of localized strain or deformation patterns that may be missed by discrete sensors. While traditional methods provide higher point-specific accuracy, their limited spatial coverage can overlook subtle changes along the structure. In terms of cost and deployment, the DFOS systems require higher initial expenses due to the need of specialized equipment and fiber installation but become more economical and efficient for large-scale or long-term monitoring, as one fiber can replace hundreds of discrete sensors. In contrast, the traditional techniques are more suitable for small-scale applications but become labor-intensive and costly when dense monitoring networks are required. These characteristics make DFOSs particularly advantageous for large-scale geotechnical and structural health monitoring projects.

## 8. Conclusions

This study presents an investigation of the field monitoring of a bored pile wall using DFOSs. Both grouting and excavation phases of the secant pile wall were considered in this monitoring study. Our conclusions and findings are as follows:

(1) The internal temperature increased by about 69 °C on average due to the cement hydration reaction inside the measurement borehole (30 min after cement grouting was completed) and a corresponding shrinkage strain of 0.5% on average was measured, due to the hardening effect of the cement slurry;
(2) The excavation depth had a significant influence on the strain distribution inside two boreholes in the monitored pile. A compressive strain distribution (inside the two opposite boreholes) of the monitored secant pile was clearly observed and increased substantially as the excavation progressed. The capping beam mounted at the pile heads exerted a substantial restrain on the induced deformation, decreasing the pile movement towards the excavation area;
(3) The displacement distribution of the pile wall was computed by both NIM and FDM. The calculated maximum displacement of the presented secant pile wall increased as the excavation depth rose, which was consistent with the curve trends observed in previous studies and was also reconfirmed by the fact that the calculated ρ between maximum lateral deflection and excavation depth equaled 0.423. A comparative study indicated that the lateral displacement of the presented pile was limited after excavation due to the high stiffness of the secant pile wall.

The use of the DFOS technology demonstrated its potential to provide high-resolution, distributed-strain data, enabling a more precise monitoring of pile behavior during construction. This will enhance the ability to assess structural performance in real time, contributing to safer and more efficient construction practices, particularly in urban excavation projects where deformation control is critical. The results also reinforce the suitability of secant pile walls as retaining structures requiring minimal deformation, offering valuable guidance for future designs. This work opens several avenues for future research. Investigating the behavior of secant pile walls in different soil types or under varying loading scenarios could expand the applicability of these findings. The long-term monitoring of pile walls under operational conditions will provide insights into their performance over time, including creep effects and cyclic loading behavior. Additionally, integrating DFOS data in advanced numerical models could improve the accuracy of soil–structure interaction predictions. Future research should also explore the application of DFOSs in other retaining structures and their potential for real-time monitoring and automated decision-making during construction.

## Figures and Tables

**Table 1:** Parameter setting of BOTDA data logger.

Equipment Parameter	Parameter Setting
Sampling resolution	0.2 m
Spatial resolution	0.5 m
Reading interval	2 ns
Frequency step	2 MHz
Sensor line length	300 m

**Table 2:** Physical parameters of CDG.

Specific Gravity	Dry Density (g/cm^3^)	Plastic Limit (%)	Liquid Limit (%)	Cohesion (kPa)	Friction Angle (°)
2.6	1.75	22.7	32.8	26	34

## Data Availability

The data that support the findings of this study are available upon reasonable request.
